# Gut microbiota-derived metabolite phenylacetylglutamine inhibits the progression of prostate cancer by suppressing the Wnt/β-catenin signaling pathway

**DOI:** 10.3389/fphar.2025.1528058

**Published:** 2025-03-11

**Authors:** Jing Lv, Shengkai Jin, Yuhua Zhou, Chaowei Fu, Yang Shen, Bo Liu, Jufa Li, Menglu Li, Yuwei Zhang, Ninghan Feng

**Affiliations:** ^1^ Wuxi School of Medicine, Jiangnan University, Wuxi, China; ^2^ Department of Urology, Jiangnan University Medical Center, Wuxi, China; ^3^ Jiangnan Medical Center, Nanjing Medical University, Nanjing, China; ^4^ Medical School of Nantong University, Nantong, China

**Keywords:** phenylacetylglutamine, prostate cancer, proliferation, migration, CCNG2, Wnt/β-catenin pathway

## Abstract

**Background:**

Prostate cancer is one of the most common malignant tumors among men worldwide, and current treatments still face many challenges. Therefore, researchers are continuously seeking new therapeutic methods to improve treatment efficacy and reduce side effects. Phenylacetylglutamine (PAGln), a common metabolite of the gut microbiota, has been reported to have anti-inflammatory and anti-tumor activities.

**Methods:**

We assessed the impact of PAGln on prostate cancer using *in vitro* and *in vivo* models. Cell proliferation, migration, and invasion capabilities were evaluated through CCK8, EdU incorporation, and colony formation assays, as well as wound healing and Transwell assays. The *in vivo* anti-cancer effects of PAGln were evaluated using a BALB/c nude mouse xenograft model of prostate cancer and a lung metastatic tumor model established via tail vein injection. Molecular mechanisms were investigated through qRT-PCR and Western blot analysis.

**Results:**

PAGln inhibited the proliferation, migration, and invasion of prostate cancer (PCa) cells *in vitro* and suppressed the growth of prostate cancer *in vivo*. PAGln notably increased the mRNA levels of CCNG2 in PCa cells. Importantly, the knockdown of CCNG2 weakened the effects of PAGln on PCa cells. Mechanistic studies revealed that PAGln could promote the phosphorylation of β-catenin by upregulating CCNG2, thereby inhibiting the Wnt/β-catenin signaling pathway.

**Conclusion:**

In summary, PAGln can effectively inhibit the proliferation, migration, and invasion of PCa by upregulating CCNG2 and suppressing the Wnt/β-catenin signaling pathway. These findings suggest that PAGln may serve as a promising therapeutic agent for prostate cancer.

## 1 Introduction

Prostate cancer (PCa) ranks as the second most prevalent cancer among men worldwide and accounts for the sixth highest cause of cancer mortality in men. It is anticipated that by 2040, due to population growth and aging, the incidence of PCa will soar to nearly 2.3 million new cases annually, with approximately 740,000 deaths projected (Siegel et al., 2024). Although approximately 80% of PCa patients are diagnosed at a localized stage, there is still a certain proportion of patients diagnosed with advanced PCa, who have a relatively lower 5-year survival rate (Siegel et al., 2022). Patients with advanced PCa initially respond well to androgen deprivation therapy (ADT); however, despite suppressed testosterone levels, some patients inevitably progress to castration-resistant prostate cancer (CRPC) after a period of ADT treatment, which is the main cause of death for the majority of PCa patients (Chandrasekar et al., 2015; Kirby et al., 2011). In recent years, various treatment methods have been proven to have the potential to slow down the progression of PCa and improve the prognosis of CRPC patients. However, the advancement of CRPC treatment is hindered by the emergence of drug resistance and the associated toxicities of treatments (Turco et al., 2022; Cai et al., 2023; Teo et al., 2019). Therefore, given the limitations in the treatment options for CRPC, there is an urgent need in the field of PCa therapy to develop innovative treatments and explore new therapeutic targets. The discovery of these new targets is expected to overcome the current constraints of treatment, significantly improving patients’ survival rates and enhancing their quality of life.

In recent years, the role of gut microbiota in PCa has attracted increasing attention. Studies have demonstrated that gut microbiota may influence the development and progression of PCa by affecting inflammation, immune responses, and hormone metabolism through its metabolites, such as short-chain fatty acids (SCFAs) (Matsushita et al., 2023). Phenylacetylglutamine (PAGln) is a metabolite produced from dietary Phenylalanine through the metabolism of the gut microbiota (Krishnamoorthy et al., 2024). Specifically, gut microbes convert Phenylalanine derived from dietary proteins into Phenylpyruvic acid (PPY). Subsequently, PPY is transformed into Phenylacetic acid (PAA) through two pathways: one involves the catalyzation by Phenylpyruvate ferredoxin-oxidoreductase (PPFOR), which oxidizes and decarboxylates PPY to form Phenylacetyl-CoA, leading to the production of PAA; the other involves Phenylpyruvate decarboxylase (PPDC) catalyzes the non-oxidative decarboxylation of PPY into Phenylacetaldehyde. This compound is then further converted into PAA (Zhu et al., 2023). After PAA is absorbed into the portal circulation, hepatic and renal enzymes of the host catalyze the combination of PAA with Glutamine to form PAGln (Nemet et al., 2020). Recently, PAGln has emerged as a research hotspot due to its potential association with cardiovascular diseases. For example, the study by Nemet et al. demonstrated that PAGln enhances platelet reactivity and promotes thrombosis by activating adrenergic receptors, primarily α2A, α2B, and β2, thereby significantly increasing the risk of cardiovascular events such as myocardial infarction and stroke (Nemet et al., 2020). Additionally, several studies have indicated a potential link between PAGln and certain types of cancer. Zeleznik et al. found that elevated plasma levels of PAGln were associated with a reduced risk of breast cancer in premenopausal women (Zeleznik et al., 2021). Furthermore, Wang et al. showed that PAGln could inhibit the growth of metastatic lung tumors in mice by suppressing NF-κB activation (Wang et al., 2012). In the context of PCa, Reichard et al. conducted a case-control study and found that the serum PAGln levels were elevated in lethal PCa patients compared to men who had never been diagnosed with PCa and PCa patients who had not died during the observation period of the study (Reichard et al., 2022). Ren et al., using untargeted metabolomics, observed that the expression of PAGln in PCa tissues (PCT) was significantly lower than in adjacent non-cancerous tissues (ANT) (Ren et al., 2016). Despite these findings, the precise relationship between PAGln and PCa remains unclear and warrants further investigation.

CCNG2 (Cyclin G2), a significant cyclin family member, exhibits nucleotide sequence similarity with Cyclin G1. CCNG2 mRNA expression oscillates periodically across the cell cycle, reaching its peak during the late S phase (Horne et al., 1996). CCNG2 functions as a negative cell cycle regulator in various cancers, inhibiting cell cycle progression (Zhang et al., 2018; Bi et al., 2021; Gao et al., 2018; Qiao et al., 2018). For instance, CCNG2 can promote cell cycle arrest in breast cancer and is closely associated with patient survival rates (Zimmermann et al., 2016). Cui et al.'s research shows that CCNG2 expression is diminished in PCa tissues, and its overexpression can suppress the proliferation of PC-3 cells (Cui et al., 2014a). Despite these findings, the role and molecular mechanisms of CCNG2 in PCa remain understudied, necessitating further in-depth research.

In this study, we have elucidated the impact of PAGln on the proliferation, migration and invasion of human PCa cells and investigated the regulatory relationship between PAGln and CCNG2, as well as the influence of CCNG2 on the proliferation, migration and invasion of PCa cells. For the first time, we reveal that PAGln modulates PCa cell behavior by regulating CCNG2. Additionally, our findings suggest a link between PAGln and the Wnt/β-catenin signaling pathway, identifying new potential biomarkers and therapeutic targets for PCa treatment.

## 2 Materials and methods

### 2.1 Ethical statement and tissue collection

This study was approved by the Ethics Committee of Wuxi No. 2 Hospital affiliated with Nanjing Medical University (2022-Y-80). The peripheral blood samples from PCa and non-PCa patients were obtained from Wuxi No. 2 Hospital. The PCa tissues and their matched adjacent non-tumor tissues were obtained from patients at Wuxi No. 2 Hospital and all participants have signed informed consent forms. All patients received no endocrine therapy before surgery, and all patients underwent radical prostatectomy. The tissues were immediately preserved in liquid nitrogen.

### 2.2 Reagents

BeyoClick™ EdU Cell Proliferation Kit with Alexa Fluor 488 and Triton X-100 was procured from Beyotime (Shanghai, China). FastPure Cell/Tissue Total RNA Isolation Kit V2, HiScript III RT SuperMix, and ChamQ Universal SYBR qPCR Master Mix Q711-02 were all acquired from Vazyme (Nanjing, China). Cell Counting Kit-8 was obtained from DOJINDO (Shanghai, China). RIPA lysis buffer was purchased from Beyotime (Shanghai, China). Protease Inhibitor Cocktails (Cat. HY-K0010) were sourced from MedChemExpress (Shanghai, China). Phosphatase Inhibitor Cocktails (Cat. ab201113) were obtained from Abcam (Shanghai, China). Phenylacetylglutamine was purchased from Selleck (CAS: 28047-15-6, Shanghai, China). Anti-CCNG2 Monoclonal antibody was purchased from Santa Cruz Biotechnology (1F9-C11, Shanghai, China), Anti-CCNG2 Polyclonal antibody (Cat. ab203314) was purchased from Abcam (Shanghai, China). Antibodies against β-catenin (Cat. 51067-2-AP), phospho-β-catenin (Ser37) (Cat. 28776-1-AP), TCF7 (Cat. 14464-1-AP), c-Myc (Cat. 10828-1-AP), ADRB2 (Cat. 29864-1-AP), Ki67 (Cat. 27309-1-AP) and Beta Actin (Cat. 66009-1-Ig) were purchased from Proteintech Company (Wuhan, China). Secondary antibodies (Cat. SA00001-1and Cat. SA00001-2) were purchased from Proteintech Company (Wuhan, China).

### 2.3 Cell culture and transfection

Normal prostate epithelial cell line (RWPE-1) and human PCa cell lines (PC3, DU145, LNCaP and 22RV1) were purchased from the American Type Culture Collection (ATCC, United States). F-12K medium was procured from Boster (Wuhan, China), while the K-SFM, RPMI-1640, EMEM medium were obtained from Gibco (Shanghai, China). Fetal bovine serum (FBS, Cat. CTCC-002-071-50) was acquired from Meisen CTCC (Zhejiang, China). Penicillin/Streptomycin was purchased from Gibco (Shanghai, China). RWPE-1 cells were cultured in K-SFM medium, PC3 cells in F-12K medium, LNCaP and 22RV1 cells were cultured in RPMI-1640 medium and DU145 cells in EMEM medium. All media were supplemented with 10% fetal bovine serum (FBS) and 1% penicillin/streptomycin, and the cells were cultured under conditions of 37°C and 5% CO_2_. All cell lines used in this study were tested and authenticated by DNA sequencing using the short tandem repeats (STR) method (ABl 3730XL Genetic Analyzer) and tested for the absence of *mycoplasma* contamination (MycoAlert). The latest test was in December 2022.

The siRNA targeting CCNG2 and the corresponding si-NC were purchased from Tsingke Biotech (Beijing, China) and transfected into PC3 and DU145 cell lines using Lipofectamine 3,000 (Thermo Fisher Scientific, Shanghai, China), according to the manufacturer’s instructions. The cells were incubated for 48 h post-transfection to ensure sufficient knockdown of the target gene. Transfection efficiency was determined using western blotting and qRT-PCR. The sequences can be seen in Supplementary Table S2.

### 2.4 Cell counting kit-8 (CCK8) and colony formation assay

The designated cells were seeded evenly in 96-well plates at a density of 3 × 10³ cells per well and incubated at 37°C in a 5% CO_2_ atmosphere for 24 h. The cells were then treated with varying concentrations of PAGln (0, 2, 4, 8, 10, and 12 mM) and further incubated for 48 h. Subsequently, 10 µL of CCK-8 reagent was added to each well according to the manufacturer’s instructions, and the absorbance at 450 nm was measured using a Multiskan FC microplate reader after 1 h of incubation. In the time-course experiment, the designated cells were seeded in 96-well plates at a density of 3 × 10³ cells per well and incubated at 37°C in a 5% CO_2_ atmosphere for 24 h. The cells were then treated with 10 mM PAGln, and the above CCK-8 detection protocol was repeated at different time points (0, 24, 48, 72, and 96 h) to record the absorbance values. Each experimental group was independently repeated three times.

For the colony formation assay, 1,000 cells/well of the indicated cells were seeded into a 6-well plate and cultured in an atmosphere of 5% CO2 at 37°C for 24 h. Then, the cells were then treated with PAGln (0 and 10 mM) and incubated for 10 days. Cells were then fixed with 4% paraformaldehyde for 30 min and stained with 0.1% crystal violet for 20 min at room temperature. The number of colonies was counted using ImageJ software (version 1.54f). Each experimental group was independently repeated three times.

### 2.5 EdU cell proliferation assay

BeyoClick™ EdU Cell Proliferation Kit with Alexa Fluor 488 was employed for the assessment of cellular multiplication. Briefly, about 1 × 10^5^ cells were cultured in 6-well plates for 24 h. Subsequently, cells were incubated with EdU for 2 h, fixed with 4% paraformaldehyde for 15 min, and permeated with 0.3% Triton X-100 for another 15 min. The cells were incubated with the Click Reaction Mixture for 30 min at room temperature in a dark place and then incubated with 1× Hoechst 33,342 for 10 min to counterstain the nucleus. Fluorescence images were captured using an Olympus CKX53 microscope (×100 magnification) (Tokyo, Japan). Each experimental group was independently repeated three times.

### 2.6 Wound-healing assay

PC3 and DU145 cells were seeded onto 6-well plates at a density of 1 × 10^5^ cells per well and grown until a confluent monolayer was formed. A sterile 200 μL pipette tip was used to create a straight scratch across the monolayer of cells. The detached cells were gently washed away with PBS, and serum-free medium was added to minimize the effect of cell proliferation. Images of the scratch area were captured at 0 h and 24 h using an Olympus CKX53 microscope (×100 magnification) (Tokyo, Japan). The scratch width or cell migration distance was measured using ImageJ software (version 1.54f) to calculate the cell migration rate. Each experimental group was independently repeated three times.

### 2.7 Transwell assay

Cell migration and invasion assays were performed using 24-well Transwell cell culture chambers (Corning, United States) with or without Matrigel (Corning). Cells (1 × 10^5^) suspended in 200ul of medium without FBS were added to the top of inserts, and 600ul of medium containing 10% FBS was added to the bottom chamber. After incubation at 37°C and 5% CO_2_ for 24 h, the cells on the lower surface of the chamber were stained with crystal violet for 30 min. The migrated cells per chamber were counted in three randomly selected fields, and independent experiments were performed in triplicate. Images were acquired using an Olympus CKX53 microscope (×100 magnification) (Tokyo, Japan), and cells were counted by ImageJ software (version 1.54f). Each experimental group was independently repeated three times.

### 2.8 Total RNA extraction and quantitative real-time PCR (qRT–PCR)

Total RNA was isolated from cells and tissues with FastPure Cell/Tissue Total RNA Isolation Kit V2 and reverse-transcribed into complementary DNAs (cDNAs) with HiScript III RT SuperMix. The cDNAs were amplified based on the standard qPCR protocol with ChamQ Universal SYBR qPCR Master Mix in an Applied Biosystems 7,500 Fast Real-Time PCR System (Thermofisher, US). Quantitative PCR was conducted at 95°C for 30 s, followed by 40 cycles of 95°C for 10 s and 60°C for 30 s. GAPDH was used as the internal control and the results were calculated using 2^−ΔΔCT^ method. Each experimental group was independently repeated three times. The sequences of primers can be seen in Supplementary Table S1.

### 2.9 Western blot assay

RIPA lysis buffer containing protease inhibitor and Phosphatase Inhibitor was used to extract total cellular and tissue protein. Protein concentration was determined with BCA Protein Assay Kit (Beyotime, China). ColorMixed Protein Marker (Thermofisher, US) was applied as a protein size marker. Total proteins were separated by 10% sodium dodecyl sulfate–polyacrylamide gel electrophoresis and then transferred to a polyvinylidene difluoride (PVDF) membrane (3010040001, Roche, Germany). The PVDF membrane was then blocked with 5% milk for 2 h followed by incubation with the indicated primary antibody overnight at 4°C. After incubation with the corresponding secondary antibody at room temperature for 1.5 h, the protein bands were visualized using enhanced chemiluminescence (Tanon, China). For all WB images, quantitative density analyses were performed using ImageJ software (version 1.54f). Each experimental group was independently repeated three times.

### 2.10 Enzyme-linked immunosorbent assay (ELISA)

The PAGln enzyme-linked immunosorbent assay (ELISA) kit was purchased from Meimian (Jiangsu, China). According to the manufacturer’s protocol, the concentration of PAGln in PCa specimens and their paired normal tissues was detected using an ELISA kit. Subsequently, the absorbance values were measured at 450 nm using a Multiskan FC microplate reader. Each experimental group was independently repeated three times.

### 2.11 Immunohistochemistry (IHC)

Tissues were fixed with 4% paraformaldehyde, dehydrated, embedded in paraffin and sectioned at 4 μm. The sections were deparaffinized, then boiled with citrate solution, and incubated with 3% H_2_O_2_ for 10 min. After antigen retrieval and blocking, the slides were incubated with the indicated antibodies at 4°C overnight. Subsequently, the slides were incubated with the corresponding secondary antibody for 30 min at room temperature and then incubated with the streptavidin peroxidase complex. Staining was performed using a 3,3-diaminobenzidine (DAB) substrate kit for peroxidase reaction and counterstained with hematoxylin. Sections were washed, dehydrated, and sealed with neutral balsam. Finally, an Olympus CKX53 microscope was used to acquire images.

### 2.12 *In vivo* experiments

This study was approved by Institutional Animal Care and Use Committee of Jiangnan University (JN.No20230830b0401115). Twenty healthy 6-week-old male BALB/c nude mice were purchased from Wukong Biotechnology (Nanjing, China).

At one time point, approximately 1 × 10^7^ PC3 cells washed with PBS were injected into the subcutaneous tissue on the right lateral side of mice. One week after tumor cell inoculation, the animals were randomly assigned to two experimental groups, each consisting of five mice. Mice in the experimental group received daily intraperitoneal injections of 200 mg/kg PAGln solution for four consecutive weeks, while those in the control group received an equal volume of physiological saline injections daily (Wang et al., 2012; Fu et al., 2023). Under general anesthesia (induced by intraperitoneal injection of sodium pentobarbital at a dose of 150 mg/kg), all mice were euthanized, and the tumors were subsequently excised for further analysis.

Similarly, at a specific time point, approximately 2 × 10^6^ PC3 cells washed with PBS were injected into the tail veins of each mouse. Two weeks post-injection, the animals were randomly divided into two experimental groups, each also consisting of five mice. Mice in the experimental group received daily intraperitoneal injections of 200 mg/kg PAGln solution for four consecutive weeks, while those in the control group received an equal volume of physiological saline injections daily. Under general anesthesia (induced by intraperitoneal injection of sodium pentobarbital at a dose of 150 mg/kg), all mice were euthanized, and the mouse lung tissues were subsequently excised for further analysis.

### 2.13 Statistical analysis

All experiments were independently conducted three times to ensure the reproducibility and statistical reliability of the results. Data are presented as the mean ± SD. Paired or nonpaired two-tailed t-tests were used for comparisons between two groups and comparison among multiple groups was achieved by analysis of One-Way ANOVA. GraphPad Prism software (version 8.0.1) was used for statistical analysis. p < 0.05 was considered significant.

## 3 Results

### 3.1 PAGln inhibits the proliferation of PCa cells

**FIGURE 1:**
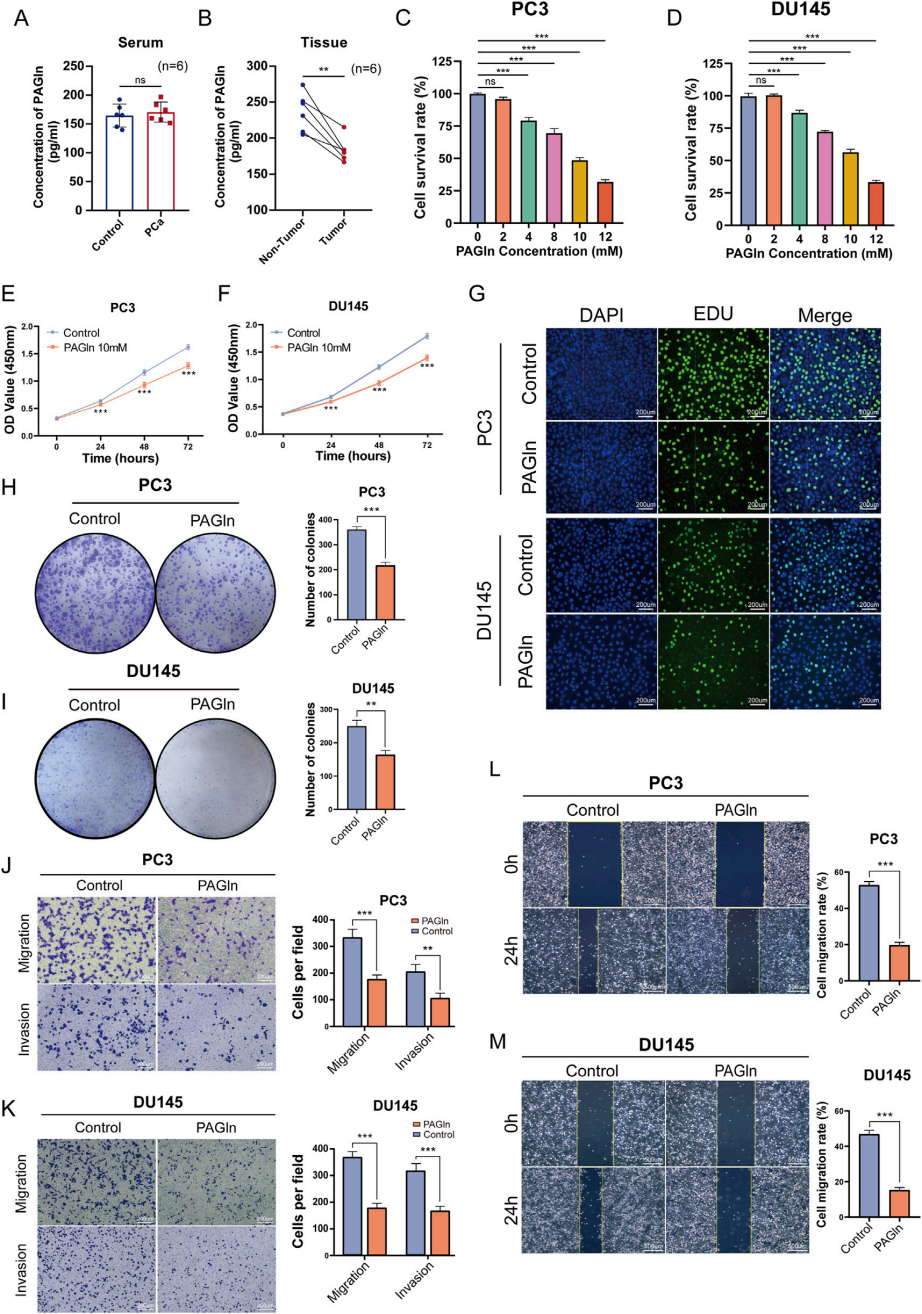
PAGln inhibits the proliferation, migration and invasion of prostate cancer cells. **(A)** PAGln concentration in the serum of prostate cancer patients and non-cancer individuals (n = 6). **(B)** PAGln concentration in tumor tissues and matched adjacent non-tumor tissues from prostate cancer patients (n = 6). **(C, D)** PC3 and DU145 cells are treated with the specified concentrations of PAGln for 48 h. Subsequently, they are subjected to a CCK-8 assay to assess their viability (n = 3). **(E, F)** PC3 and DU145 cells are treated with 10 mM PAGln for different times, and CCK-8 assays are used to measure cell viability at different times (n = 3). **(G)** The proliferation of PC3 and DU145 cells is evaluated by EdU assay (n = 3). **(H, I)** The colony formation assay is conducted 10 days after treatment with PAGln (n = 3). **(J, K)** The migration and invasion capability of prostate cancer cells after PAGln treatment is also detected by Transwell assay (n = 3). **(L, M)** A wound healing assay is used to determine the effect of PAGln on the migration of prostate cancer cells for 24 h (n = 3). Data are presented as the mean ± SD. ns P > 0.05, **P < 0.01, ***P < 0.001 compared to control, Student's t-test **(A, B, E, F, H and J–M)** and One-Way ANOVA **(B, C)**. PAGln: Phenylacetylglutamine.

Firstly, to evaluate the expression levels of PAGln in the serum of PCa patients and non-cancer patients, we collected serum samples from 6 PCa patients and six non-cancer patients, and measured the PAGln concentration in the serum samples using a human PAGln ELISA kit. The results showed that the PAGln concentrations in both groups were similar, with no statistically significant difference (*P* = 0.5799) (Figure 1A). Next, we collected tumor tissues and their paired adjacent non-tumor tissues from 6 PCa patients, and measured the PAGln concentration in the tissue samples using the same ELISA kit. The results indicated that the PAGln concentration in tumor tissues was significantly lower than that in the paired adjacent non-tumor tissues (Figure 1B). Subsequently, to assess PAGln’s impact on PCa cell proliferation, we performed a CCK-8 assay to measure the viability of PC3, DU145, LNCaP, and 22RV1 cells following treatment with various PAGln concentrations. After 48 h, PAGln inhibited the growth of both PC3 and DU145 cells in a concentration-dependent manner compared to the control group, with IC50 values of 9.7 mM and 10.38 mM, respectively (Figures 1C, D). Similarly, PAGln concentration-dependently inhibited the growth of LNCaP and 22RV1 cells (Supplementary Figures S1A, B). To evaluate PAGln’s effect on normal prostate cells, we treated human prostate epithelial cells RWPE-1 with different concentrations of PAGln. The results indicate that these concentrations had no significant effect on RWPE-1 cell growth, suggesting low toxicity to normal prostate cells (Supplementary Figure S1C). Based on these results, we selected 10 mM as the intervention concentration for subsequent experiments. We then measured cell viability at this concentration over 0, 24, 48, and 72 h using the CCK-8 assay. Findings showed a time-dependent increase in PAGln’s inhibitory effect on both PC3 and DU145 cells (Figures 1E, F), with similar results in LNCaP and 22RV1 cells (Supplementary Figures S1D, E).

We also conducted a 5-ethynyl-2-deoxyuridine (EdU) incorporation assay, commonly used to indicate DNA synthesis, to confirm PAGln’s effects on cell proliferation. The number of EdU-positive cells was lower in the PAGln-treated group compared to the control group (Figure 1G; Supplementary Figure S1F). Additionally, we verified PAGln’s impact on the colony-forming ability of PCa cells using a colony formation assay. As shown in Figure 1H, I and Supplementary Figure S1G, the number and size of colonies were significantly reduced in the PAGln-treated group compared to the control group, indicating that PAGln markedly inhibits the colony formation of PCa cells. These experimental results suggest that PAGln effectively inhibits PCa cell proliferation *in vitro*.

### 3.2 PAGln inhibits the migration and invasion of PCa cells

To investigate the effects of PAGln on the migration and invasion of PCa cells, we performed Transwell migration and invasion assays. The results showed a significant reduction in the migratory and invasive activities of PC3 and DU145 cells in the PAGln-treated group compared to the control group (Figures 1J, K). Similarly, Transwell migration assays with LNCaP and 22RV1 cells indicated that PAGln also inhibited the migratory ability of these cell lines (Supplementary Figure S1H). Additionally, a wound healing assay was conducted to further confirm PAGln’s inhibitory effect on cell migration. Results demonstrated that PAGln significantly reduced PCa cell migration compared to the control group (Figures 1L, M). Overall, these findings suggest that PAGln effectively suppresses the migration and invasion capabilities of PCa cells *in vitro*.

### 3.3 PAGln’s effects on PCa cells are independent of ADRB2

Previous studies have indicated that PAGln can act through various adrenergic receptors, including α2A, α2B, and β2 adrenergic receptors (ADRs) (Nemet et al., 2020). Additionally, the luminal cells of the prostate are highly enriched with β-adrenergic receptors, which are predominantly β2 receptors (Braadland et al., 2014). We detected the mRNA expression levels of α2A, α2B, and β2 adrenergic receptors in human prostate epithelial cells RWPE-1 and PCa cells PC3, DU145, 22RV1, LNCaP, and VCaP using q-PCR experiments. The results showed that compared to β2, the mRNA expression levels of α2A and α2B adrenergic receptors were both extremely low in all cell lines (Supplementary Figures S2A). Subsequently, we assessed the results revealed that the expression of ADRB2 was significantly higher in PC3, DU145, 22RV1, LNCaP, and VCaP cells compared to RWPE-1 (Supplementary Figures S2B, C). To investigate whether PAGln exerts its effects via ADRB2, we treated PC3 cells with the ADRB2 inhibitor ICI 118551 and the agonist isoproterenol (ISO), in conjunction with a 48-hour PAGln intervention. Cell viability was assessed using the CCK8 assay, and we found that the inhibitory effect of PAGln on PC3 cells appeared to be unaffected by ICI-118551 and ISO (Supplementary Figure S2D). Similar results were also observed in LNCaP and 22RV1 cells (Supplementary Figures S1E, F). Based on these observations, we believe that the inhibitory effect of PAGln on PCa cells may not be mediated by ADRB2.

### 3.4 PAGln promotes the expression of CCNG2

**FIGURE 2:**
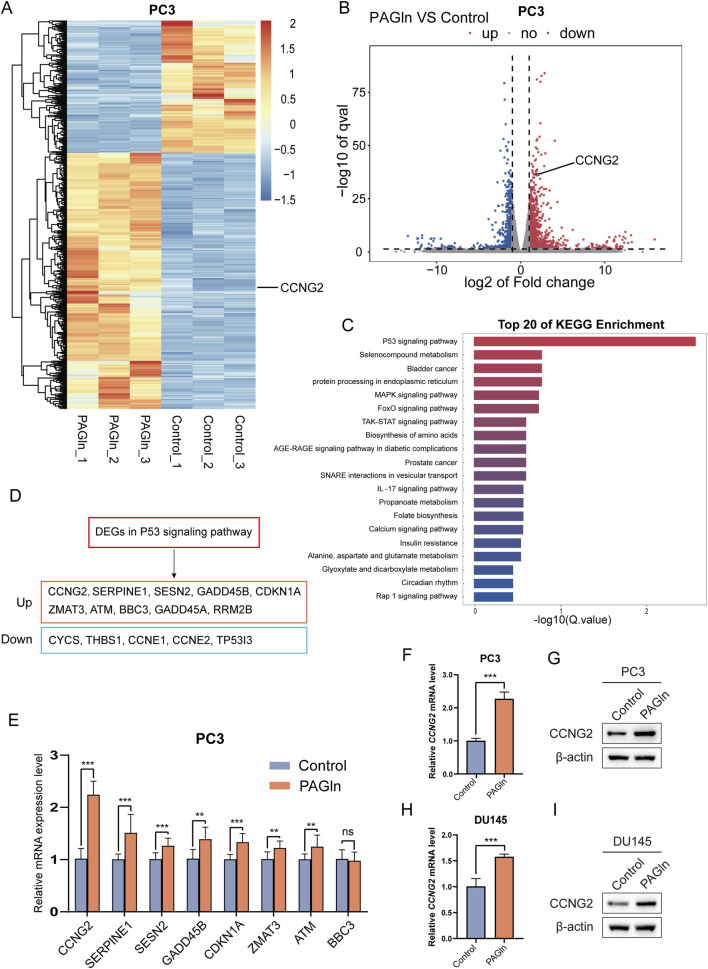
PAGln promotes the expression of CCNG2. **(A, B)** The heatmap and volcano plot illustrate the visual analysis results of the expression differences of the CCNG2 gene between PAGln-treated samples and their corresponding control samples. **(C)** The top 20 KEGG pathway enrichments of differentially expressed genes following PAGln treatment. **(D)** Differentially expressed genes that were upregulated and downregulated in the P53 signaling pathway following PAGln treatment. **(E)** The expression levels of the selected genes at the mRNA level are analyzed using quantitative PCR technology. **(F–I)** qRT-PCR and western blot analyses reveal the impact of PAGln treatment on the expression of the CCNG2 gene in PC3 and DU145 cells. Data are presented as the mean ± SD (n = 3). ns P > 0.05, **P < 0.01, ***P < 0.001 compared to control, Student’s t-test. PAGln: Phenylacetylglutamine.

To further elucidate the molecular mechanisms underlying PAGln’s inhibitory effects on PCa cell proliferation and migration, we conducted a reference-based transcriptome analysis on PC3 cells following a 48-hour PAGln treatment. Compared to the control group, 1,311 genes were upregulated and 677 genes were downregulated in the PAGln group (Supplementary Figure S3A; Supplementary Table S1). Notably, among the upregulated genes, CCNG2 was identified (Figures 2A, B). KEGG pathway enrichment analysis ranked pathways by corrected p-values (Q-values), identifying the top 20 most significant metabolic pathways. This analysis revealed a significant enrichment of differentially expressed genes in the P53 signaling pathway (Figure 2C). There are a total of 15 differentially expressed genes enriched in the p53 signaling pathway, among which 10 are upregulated genes and five are downregulated genes (Figure 2D; Supplementary Table S3). To explore the key regulatory genes that influence cellular phenotypes in response to PAGln, we ranked the differentially upregulated genes in descending order of fold change. Through q-PCR experiments, we found that CCNG2 was significantly and stably upregulated after PAGln treatment (Figure 2E). Therefore, we selected CCNG2 for further research. The q-PCR results confirmed that PAGln upregulated CCNG2 mRNA expression in PC3 and DU145 cells (Figures 2F, G). Western blot analysis further validated that PAGln increased CCNG2 protein expression in these cells (Figures 2H, I). Collectively, these results suggest that PAGln may inhibit PCa cell progression by upregulating CCNG2 expression.

### 3.5 Knockdown of CCNG2 promotes the proliferation and migration of PCa cells

**FIGURE 3:**
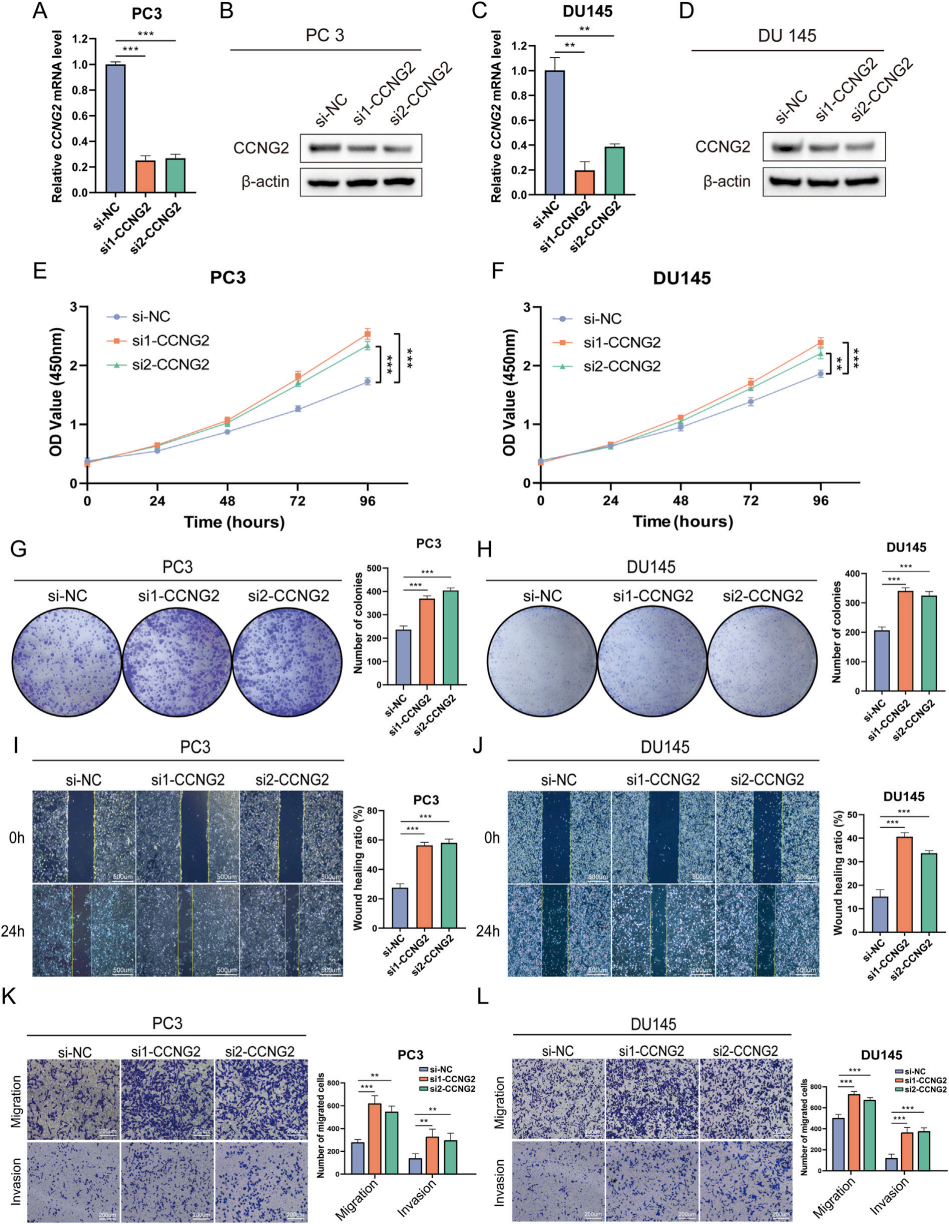
Knockdown of CCNG2 promotes the proliferation and migration of prostate cancer cells. **(A–D)** qRT-PCR and western blot analyses are performed to evaluate the knockdown efficiency of CCNG2 in PC3 and DU145 cells. **(E, F)** After CCNG2 knockdown in PC3 and DU145 cells, cell proliferation is assessed at different times using the CCK-8 assay, with absorbance measured at 450 nm. **(G, H)** The colony formation assay is conducted 10 days after knockdown of CCNG2 in PC3 and DU145 cells. **(I–L)** The wound healing, Transwell migration and invasion assays are conducted after knockdown of CCNG2 in PC3 and DU145 cells. Data are presented as the mean ± SD (n = 3). **P < 0.01, ***P < 0.001 compared to si-NC, One-Way ANOVA. si-NC: negative control of small interfering RNA (siRNA); si1-CCNG2: small interfering RNA one for CCNG2; Si2-CCNG2: small interfering RNA two for CCNG2.

To clarify the role of CCNG2 in PCa cell proliferation and migration, we performed CCNG2 knockdown in PC3 and DU145 cell lines and confirmed knockdown efficiency using q-PCR and Western blot analyses (Figures 3A–D). CCK-8 and colony formation assays revealed a significant increase in proliferation in both PC3 and DU145 cells following CCNG2 knockdown (Figures 3E–H). Additionally, wound healing and Transwell assays demonstrated that CCNG2 reduction markedly enhanced cell migration and invasion (Figures 3I–L). EdU staining further indicated a significant increase in EdU-positive cells after CCNG2 knockdown (Supplementary Figures S3B, C). Based on the results from qPCR and Western blot experiments, as well as functional assays in the PC3 and DU145 cell lines, si1-CCNG2 demonstrated better knockdown efficiency and more significant functional outcomes. Therefore, we ultimately chose si1-CCNG2 for subsequent experiments.

### 3.6 PAGln inhibits the proliferation and migration of PCa cells by promoting CCNG2 expression

**FIGURE 4:**
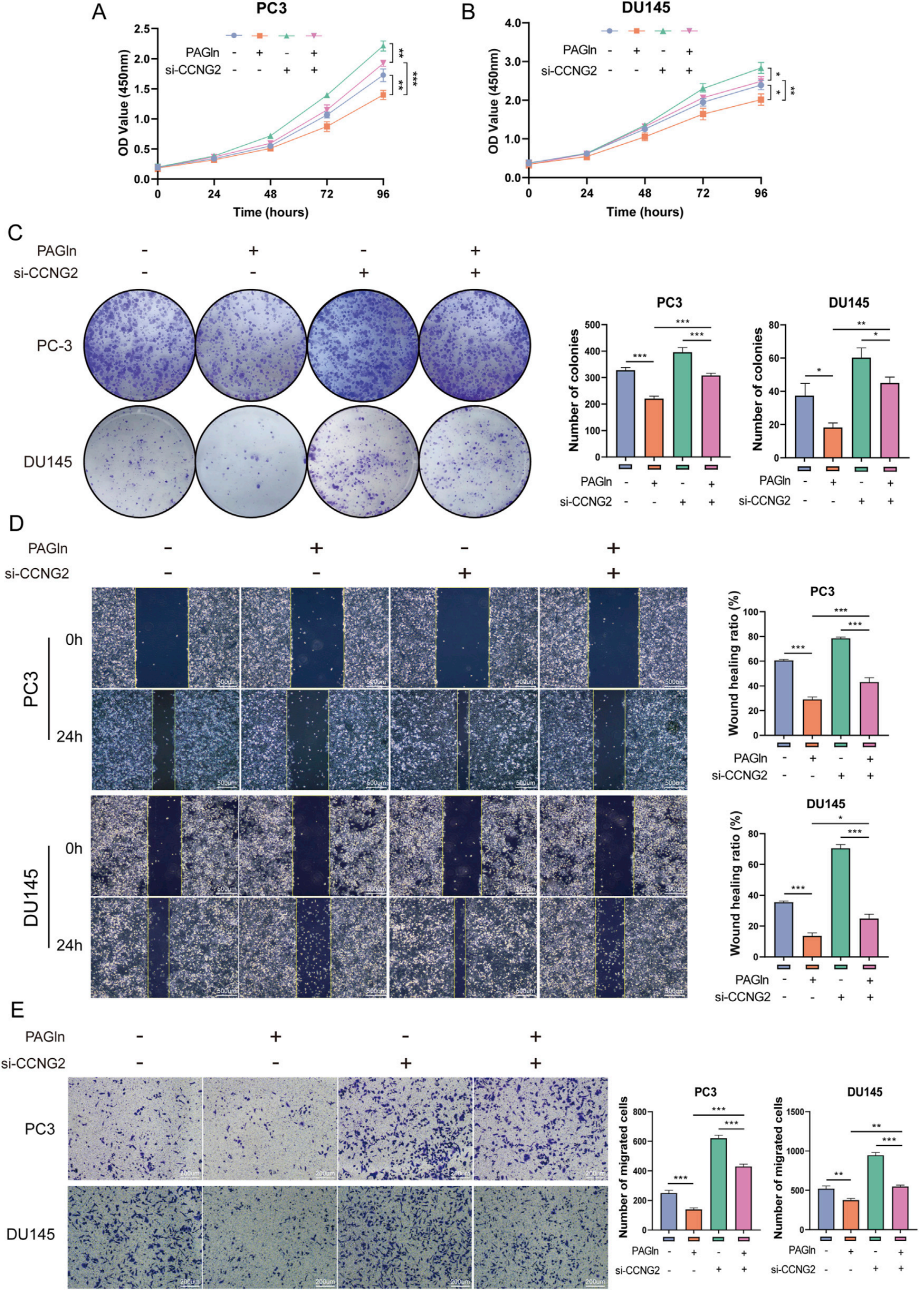
PAGln inhibits the proliferation and migration of prostate cancer cells by promoting CCNG2 expression. **(A, B)** CCK-8 assay results indicate that the proliferation of PC3 and DU145 cells, which is inhibited by PAGln treatment, is partially rescued following the knockdown of CCNG2. **(C)** Colony formation assays reveal that the inhibitory effect of PAGln treatment on the colony-forming ability of prostate cancer cells is partially reversed by the knockdown of CCNG2. **(D, E)** Wound healing and Transwell migration assays demonstrate that the PAGln-induced suppression of prostate cancer cell migration is partially rescued by the knockdown of CCNG2. Data are presented as the mean ± SD (n = 3). *P < 0.05, **P < 0.01, ***P < 0.001 compared to control, One-Way ANOVA. PAGln: Phenylacetylglutamine; si-CCNG2: small interfering RNA one for CCNG2.

To determine whether PAGln suppresses PCa cell proliferation and migration through upregulation of CCNG2, we conducted rescue experiments. CCK-8 and colony formation assays showed that CCNG2 knockdown reduced PAGln’s inhibitory effect on the proliferation of PC3 and DU145 cells (Figures 4A–C). Furthermore, wound healing and Transwell migration assays revealed that CCNG2 knockdown attenuated PAGln’s suppressive effect on cell migration (Figures 4D, E). These findings suggest that PAGln inhibits PCa cell proliferation and migration by promoting CCNG2 expression.

### 3.7 PAGln suppresses the Wnt/β-catenin signaling pathway in PC3 and DU145 cells

**FIGURE 5:**
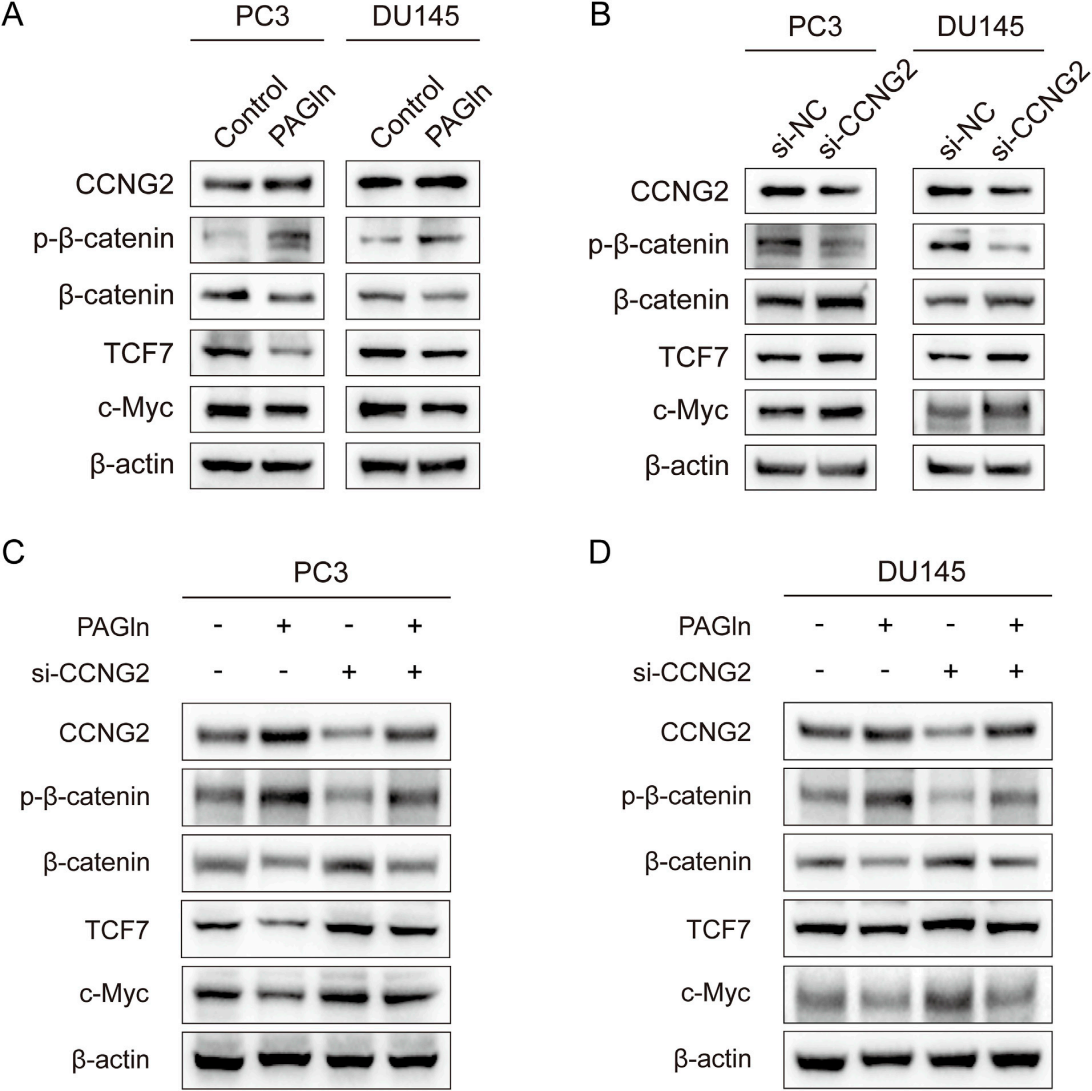
PAGln suppresses the Wnt/β-catenin signaling pathway in prostate cancer cells. **(A)** WB assays confirm that PAGln inhibit the Wnt/β-catenin pathway in PC3 and DU145 cells. **(B)** WB assays show that knockdown of the CCNG2 enhance the Wnt/β-catenin pathway. **(C, D)** WB assays indicate that the knockdown of CCNG2 attenuates the inhibitory effect of PAGln on the Wnt/β-catenin signaling pathway in PC3 and DU145 cells. PAGln: Phenylacetylglutamine; si-NC: negative control of small interfering RNA (siRNA); si-CCNG2: small interfering RNA one for CCNG2. Data presented from three independent experiments.

To explore the specific mechanisms by which CCNG2 affects cell phenotypes, we reviewed the literature and found that CCNG2 can inhibit the Wnt/β-catenin pathway through multiple mechanisms, including by reducing the nuclear translocation of β-catenin to suppress its transcriptional activity. This suggests that PAGln may regulate the activity of the Wnt/β-catenin pathway through CCNG2 as a key node (Gao et al., 2018). To validate this hypothesis, we used Western blot analysis to examine whether PAGln modulates PCa progression through the Wnt/β-catenin signaling pathway. After treating PC3 and DU145 cells with PAGln for 48 h, we assessed the expression levels of CCNG2 and key proteins in the Wnt/β-catenin pathway. The results showed that PAGln treatment upregulated CCNG2, downregulated β-catenin, and increased β-catenin phosphorylation, leading to reduced expression of downstream proteins TCF7 and c-Myc (Figure 5A). Previous studies have shown that CCNG2 can inhibit the Wnt/β-catenin signaling pathway, thereby affecting tumor progression. Our results further confirmed that CCNG2 knockdown in PC3 and DU145 cells led to an increase in β-catenin expression, a decrease in phosphorylated β-catenin, and upregulation of downstream TCF7 and c-Myc proteins (Figure 5B). To further verify whether PAGln suppresses Wnt/β-catenin signaling by upregulating CCNG2, we conducted rescue experiments. Results indicated that CCNG2 knockdown in PC3 cells significantly diminished PAGln’s inhibitory effect on the Wnt/β-catenin pathway (Figure 5C), with similar findings in DU145 cells (Figure 5D). These findings suggest that PAGln effectively suppresses Wnt/β-catenin pathway activity through upregulation of CCNG2 expression.

### 3.8 PAGln inhibited PCa growth *in vivo*

**FIGURE 6:**
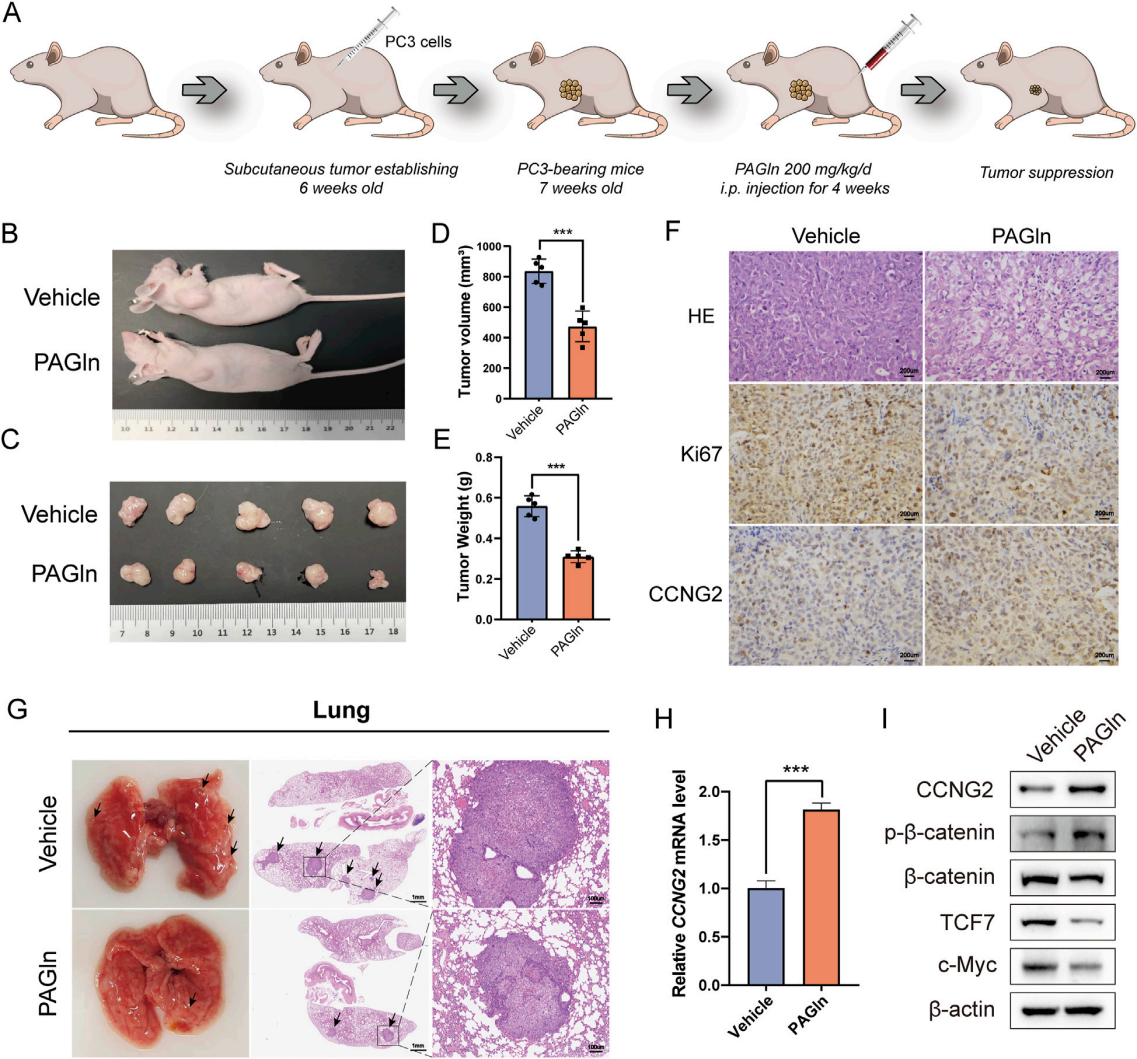
PAGln inhibits prostate cancer growth *in vivo*. **(A)** Flowchart of the *in vivo* experimental procedure. **(B)** The mice were first anesthetized, followed by photography of their overall appearance. **(C)** Subsequently, the mice are humanely euthanized, and the tumor tissues are excised and photographed for documentation (n = 5). **(D, E)** The volume and weight of tumors in the PAGln-treated groups are significantly lower than those in the vehicle group (n = 5). **(F)** HE-staining shows the histological architecture of the tumors in the mice. Immunohistochemical analysis is performed to evaluate the expression levels of Ki67 and CCNG2 in tumor tissues following PAGln treatment (×100). **(G)** HE-staining demonstrated the size and number of pulmonary metastatic nodules. **(H)** The expression of CCNG2 mRNA in tumor tissues of two groups of mice was detected through q-PCR experiments (n = 3). **(I)** WB experiments have confirmed that PAGln suppresses the Wnt/β-catenin signaling pathway in mice tumor tissues (n = 3). Data are presented as the mean ± SD. ***P < 0.001 compared to vehicle, Student’s t-test. Vehicle: intervention with physiological saline; PAGln: Phenylacetylglutamine.

To evaluate the potential of PAGln to inhibit tumor growth *in vivo*, we conducted a series of animal experiments. First, PC3 cells were injected into 6-week-old BALB/c nude mice to establish a subcutaneous xenograft tumor model. One week post-injection, PAGln was administered to the experimental group via daily intraperitoneal injections for 28 consecutive days (Figure 6A). Based on efficacy and safety considerations, we selected a concentration of 200 mg/kg for the study. Our results showed that, compared to the vehicle control group, the PAGln-treated group exhibited a significantly slower tumor growth rate (Figures 6B, C). Additionally, both tumor volume and weight were markedly reduced in the PAGln group compared to the control group (Figures 6D, E). Hematoxylin and eosin (HE) staining of tumor tissues showed that PAGln-treated tumors displayed a more spongy texture with a significantly reduced number of tumor cells relative to the vehicle control group. Immunohistochemical analysis further revealed a decrease in Ki-67 expression and an increase in CCNG2 expression in the PAGln-treated group (Figure 6F). Next, we established a mouse lung metastasis model by injecting PC3 cells via the tail vein, followed by intraperitoneal administration of PAGln at 200 mg/kg for 28 consecutive days to observe its effect on PCa metastasis. The results showed that PAGln treatment significantly reduced lung colonization by PC3 cells, with fewer and smaller tumor nodules compared to the control group (Figure 6G). In conclusion, these findings suggest that PAGln effectively inhibits both the growth and metastasis of PCa *in vivo*.

To assess PAGln’s effect on the Wnt/β-catenin signaling pathway *in vivo*, we conducted q-PCR analysis of CCNG2 expression in subcutaneous tumor tissues. Results indicated a significant upregulation of CCNG2 in PAGln-treated tumors (Figure 6H). Additionally, compared to the vehicle control group, PAGln treatment increased phosphorylated β-catenin levels and decreased the expression of downstream proteins TCF7 and c-Myc, suggesting that PAGln suppresses the Wnt/β-catenin pathway *in vivo* (Figure 6I). In summary, PAGln inhibits PCa growth and metastasis *in vivo* by upregulating CCNG2 expression and suppressing the Wnt/β-catenin signaling pathway.

## 4 Discussion

Approximately 99% of the human body’s microorganisms reside in the gut, where they are subject to a multitude of external influences such as the environment, diet, and the use of antibiotics. An imbalance in the gut microbiota can precipitate a variety of diseases (Valdes et al., 2018). Among these factors, lifestyle choices, particularly dietary habits, exert a significant and enduring impact on the gut microbiota. A diet high in fat has been shown to promote the growth of PCa, potentially due to the disruption of the gut microbiota caused by such a diet (Matsushita et al., 2022). Recent studies utilizing 16S rRNA gene sequencing on fecal samples from individuals with PCa and mice have uncovered a range of correlations between shifts in the gut microbiome and the development of PCa (Liss et al., 2018; Golombos et al., 2018; Sfanos et al., 2018; Huang et al., 2021). These findings underscore the significant role that the gut microbiota has in the initiation, progression, and metastasis of PCa (Matsushita et al., 2023). Imbalances within the gut microbiota can erode the intestinal mucosa, undermining its structural integrity and elevating intestinal permeability, resulting in a phenomenon referred to as “leaky gut.” This condition facilitates the migration of gut microbiota and their metabolites into the systemic circulation, which in turn can have an impact on organs located far from the gastrointestinal tract (Kinashi and Hase, 2021). A large number of studies have shown that metabolites produced by gut microbiota, such as SCFAs and equol, can influence the occurrence and progression of PCa (Mirzaei et al., 2021; Itsumi et al., 2016; Lv et al., 2024).

PAGln is a recently discovered gut microbiota-derived metabolite, with current research primarily focused on its effects on cardiovascular and cerebrovascular diseases. In a study by Nemet and colleagues utilizing non-targeted metabolomic profiling, it was found that PAGln facilitates the formation of blood clots and heightens the risk of cardiovascular incidents by activating platelets and engaging with adrenergic receptors, including α2A, α2B, and β2 adrenergic receptors (Nemet et al., 2020). Fu and colleagues have discovered that PAGln promotes cardiac inflammation and fibrosis in mice, thereby increasing the incidence of ventricular arrhythmias in mice with heart failure, through the activation of the TLR4/AKT/mTOR signaling pathway (Fu et al., 2023). Additionally, PAGln has also been associated with neurological disorders (Cirstea et al., 2020; Gonzalez-Dominguez et al., 2016), diabetes (Urpi-Sarda et al., 2019), chronic kidney disease (Poesen et al., 2016), Crohn’s disease (Feng et al., 2023), and obesity (Elliott et al., 2015). In addition, PAGln has been reported to exhibit anti-inflammatory and anti-tumor activities. Research by Hazekawa et al. indicates that PAGln can exert anti-inflammatory activity by inhibiting the production of inflammatory cytokines and the expression of inflammatory proteins (Hazekawa et al., 2018). A case-control study by Hang et al. found a negative correlation between PAGln and advanced colorectal adenomas (at least one adenoma with a diameter of ≥10 mm or with advanced histological characteristics, such as tubulovillous, villous, or high-grade dysplasia features) and high-risk serrated polyps (Hang et al., 2022). Zeleznik et al. assessed the association between circulating amino acids and amino acid-related metabolites with the risk of breast cancer in pre-menopausal women, discovering that high levels of PAGln in plasma were negatively correlated with the risk of breast cancer in pre-menopausal women (Zeleznik et al., 2021). Wang et al. established a lung cancer metastasis model by injecting C57BL6 mice with Lewis lung carcinoma cells via the tail vein. They discovered that after 10 days of intraperitoneal PAGln injections, pulmonary inflammation was reduced by inhibiting the activation of NF-κB. This intervention effectively suppressed the development of lung tumors (Wang et al., 2012). In our study, we report that PAGln inhibits the growth and metastasis of PCa cells, and we also demonstrate that CCNG2 and the Wnt/β-catenin pathway may be involved in the anticancer effects of PAGln on PCa.

In this study, we first analyzed the levels of PAGln in the serum of 6 PCa patients and 6 non-cancer patients. The results showed that, although the PAGln concentration was slightly higher in the serum of PCa patients compared to the non-cancer group, the difference was not statistically significant. Subsequently, we measured the PAGln concentration in tumor tissues and matched adjacent non-tumor tissues from 6 pairs of PCa patients and observed that the PAGln concentration in PCa tissues was significantly lower than that in non-tumor tissues. Similarly, a study by Ren et al. (2016) using untargeted metabolomics demonstrated that PAGln expression in PCa tissues was significantly lower than in adjacent non-tumorous tissues, which aligns with our findings. Furthermore, through cellular and animal experiments, we confirmed that PAGln can inhibit the growth and metastasis of PCa. However, Reichard et al. conducted a case-control study and found that the serum PAGln levels were elevated in lethal PCa patients compared to men who had never been diagnosed with PCa and those who had not died during the observation period of the study (Reichard et al., 2022). This discrepancy we believe are caused by several factors. First, in the study by Reichard et al., the control group included PCa patients who were still alive during the observation period, whereas our study focused on PCa patients and non-cancer controls. Second, since PAGln is primarily metabolized by the gut microbiota, differences in dietary habits and gut microbiota composition across different populations could contribute to variations in PAGln concentrations. Third, the tumor tissue and its immune microenvironment (such as immune cells, intratumoral microbiota, pH, and enzymatic activity) may affect the transport of PAGln from the circulatory system into the tumor. Lastly, in the animal experiments, we administered PAGln via intraperitoneal injection at a daily dose of 200 mg/kg, which significantly increased the PAGln concentration in the blood within a short period. We hypothesize that the elevated circulating PAGln levels might counteract the effects of the tumor microenvironment, thereby increasing PAGln levels within the tumor tissue and exerting its inhibitory effect. This result is consistent with the effects observed in our cell experiments, where we used 10 mM PAGln for intervention. These factors may collectively explain the observed phenomenon.

Previous studies have confirmed that PAGln can exert its effects by interacting with the adrenergic receptors α2A, α2B, and β2 in platelets. In our research, we examined the expression of these adrenergic receptors in prostate epithelial cells and PCa cells. The results indicated that the expression levels of α2A and α2B were extremely low in all cell lines, while the expression of the β2 receptor was generally higher in PCa cells than in normal prostate epithelial cells. However, our further research revealed that the inhibitory effect of PAGln on PCa cells does not seem to depend on the β2 adrenergic receptor. CCNG2 is a member of the cyclin G family and is recognized as an unconventional cyclin. Unlike other cyclins that promote cell cycle progression, CCNG2 is more highly expressed in cells with halted cell cycles and in terminally differentiated cells, playing a negative regulatory role in the cell cycle (Horne et al., 1996; Horne et al., 1997; Arachchige et al., 2006). CCNG2 has been reported in various types of tumors as a potential tumor suppressor and holds promise as a prognostic biomarker (Zhang et al., 2018; Bi et al., 2021; Gao et al., 2018; Qiao et al., 2018). CCNG2 is upregulated in macrophages by IFN-γ, which enhances the chemotaxis of cytotoxic T lymphocytes (CTLs) and inhibits angiogenesis to suppress tumor growth (Liu et al., 2022). Furthermore, a notable disparity in CCNG2 levels has been observed between neoplastic and healthy tissues (Cui et al., 2014b; Sun et al., 2014). Cui et al.'s research indicates that the expression of CCNG2 is reduced in PCa tissues and is significantly correlated with lymph node metastasis, clinical staging, and Gleason score (Cui et al., 2014a). This suggests that the loss of CCNG2 may contribute to the development of PCa. However, little is known about the biological function of CCNG2 in PCa. In our study, we observed that PAGln can significantly increase the expression levels of the CCNG2 protein in PCa cells. Furthermore, we found that knocking down the expression of CCNG2 can promote the proliferation, migration, and invasive capabilities of PCa cells. By conducting rescue experiments, we confirmed that PAGln inhibits the progression of PCa by upregulating the expression of CCNG2.

The classical Wnt/β-catenin signaling pathway is closely associated with the pathogenesis of various cancers, as it can regulate cell proliferation, differentiation, migration, and apoptosis (Pai et al., 2017). Activation of the Wnt/β-catenin pathway has been shown to affect the proliferation, migration, and epithelial-mesenchymal transition of PCa cells (Kypta and Waxman, 2012). Therefore, targeting the WNT complex on the cell membrane or blocking β-catenin signaling may have potential value in the prevention and treatment of PCa (Murillo-Garzon and Kypta, 2017; Krishnamurthy and Kurzrock, 2018). Consequently, we investigated the impact of PAGln on the Wnt/β-catenin signaling pathway in PCa cells. Our study observed that PAGln promoted the phosphorylation of β-catenin while reducing its expression and inhibiting its transcriptional activity. Phosphorylated β-catenin is unable to enter the nucleus to bind with downstream transcription factors and exert transcriptional regulation. We also observed a decrease in the expression of downstream transcription factors TCF7 and target gene c-Myc proteins. Thus, our research demonstrates that PAGln inhibits the Wnt/β-catenin signaling pathway, thereby suppressing the proliferation and migration of PCa cells.

There are reports in the literature that CCNG2 has an inhibitory effect on the Wnt/β-catenin signaling pathway. For instance, a study by Bernaudo et al. found that CCNG2 can effectively suppress the epithelial-mesenchymal transition of ovarian cancer cells by inhibiting Wnt/β-catenin signal transduction (Bernaudo et al., 2016). Further corroborating this, a study by Gao et al. confirmed that CCNG2 can inhibit the growth and metastasis of gastric cancer both *in vitro* and *in vivo*, an effect closely related to its suppression of the Wnt/β-catenin signaling pathway (Gao et al., 2018). Our research observed that reducing the expression level of CCNG2 would promote the activation of the Wnt/β-catenin signaling pathway, characterized by decreased levels of phosphorylated β-catenin and increased expression levels of TCF7 and c-Myc proteins. It is particularly noteworthy that in PCa cells treated with PAGln, reducing the expression of CCNG2 would weaken the inhibitory effect on the Wnt/β-catenin signaling pathway. Ultimately, our study results indicate that PAGln inhibits the development of PCa by increasing the expression level of CCNG2 and suppressing the Wnt/β-catenin signaling pathway. Nevertheless, the specific mechanism by which CCNG2 affects the classical Wnt/β-catenin signaling pathway is still unclear and requires further elucidation in future research.

There are still some limitations in the current research. First, the limited number of clinical samples in our study may restrict the generalizability and statistical significance of the findings. Future studies should consider increasing the sample size to more comprehensively assess the differential expression of PAGln in PCa patients and non-cancer populations. Secondly, in our study, the intervention concentration of PAGln is relatively high, which indicates that there are still certain limitations to the application of PAGln as a clinical drug in the future. Future research may consider exploring methods to enhance the effectiveness of PAGln, such as its use in combination with other drugs or in conjunction with immunotherapy. Thirdly, the potential impacts of PAGln on the cardiovascular system and other vital organs after intraperitoneal injection in animal experiments have not yet been monitored. Future studies should investigate the specific effective concentration of PAGln in animals and minimize its adverse effects on other systems to ensure the efficacy and safety of the treatment. Fourth, whether the role of PAGln within tumor tissue is influenced by the tumor microenvironment, and whether intratumoral microbes participate in the metabolism of PAGln, are questions that warrant further investigation. Lastly, this study has not yet explored the specific molecular mechanisms by which PAGln upregulates CCNG2. Future research will focus on uncovering the detailed mechanisms through which PAGln upregulates CCNG2 via specific signaling pathways or regulatory factors, and further investigate the role of this process in the onset and progression of PCa.

In summary, our study indicates that PAGln can inhibit the proliferation, migration and invasion of PCa cells *in vitro* and suppress tumor growth *in vivo*. The underlying mechanism is that PAGln induces the upregulation of CCNG2, which in turn leads to increased phosphorylation of β-catenin, ultimately inhibiting the Wnt/β-catenin signaling pathway. These findings suggest that PAGln and CCNG2 have potential applications in the early diagnosis, disease monitoring, and targeted therapy of PCa.

## Data Availability

The datasets presented in this study can be found in the NCBI Sequence Read Archive (SRA) under the BioProject ID PRJNA1206841. The data can be accessed at the following link: https://www.ncbi.nlm.nih.gov/bioproject/PRJNA1206841.
