# Supplementary material for: Gut microbiota-derived metabolite phenylacetylglutamine inhibits the progression of prostate cancer by suppressing the Wnt/β-catenin signaling pathway
Source: Front Pharmacol. 2025 Mar 11;16:1528058. doi: 10.3389/fphar.2025.1528058 (PMC11932994; doi:10.3389/fphar.2025.1528058)
Supplement: Supplementary file 2 [file Table1.docx]

**Gut Microbiota-Derived Metabolite Phenylacetylglutamine Inhibits the Progression of Prostate Cancer by Suppressing the Wnt/β-Catenin Signaling Pathway**


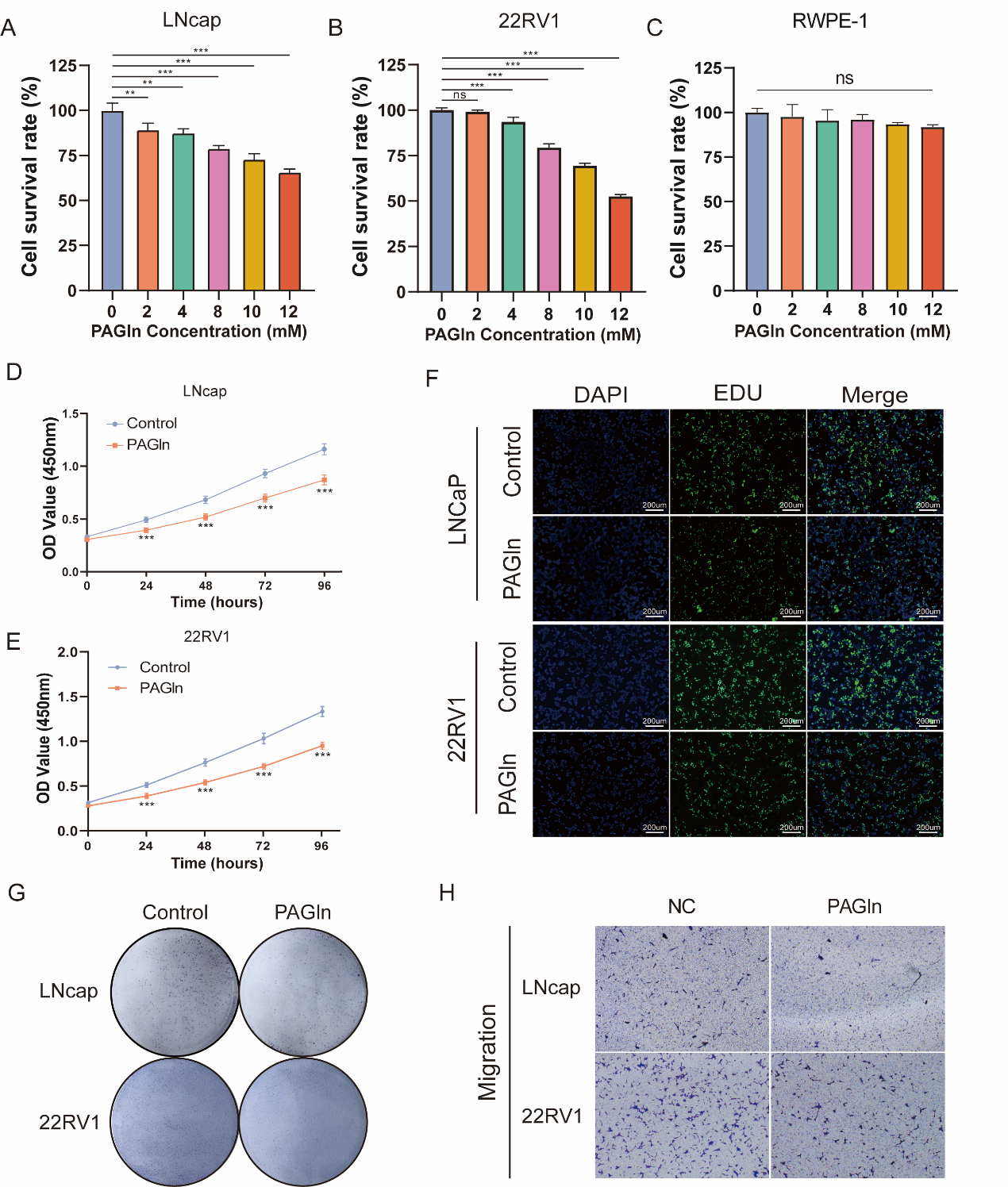


**Supplemental Figure 1.** PAGln inhibits the proliferation and migration of prostate cancer cells. (**A, B**) LNCaP and 22RV1 cells are treated with the specified concentrations of PAGln for 48 hours. Subsequently, they are subjected to a CCK-8 assay to assess their viability. (**C**) RWPE-1 cells are treated with different concentrations of PAGln for 48 hours, Cell viability is determined by CCK-8 assay. (**D, E**) LNCaP and 22RV1 cells are treated with 10mM PAGln for different times, and CCK-8 assays are used to measure cell viability at different times. (**F**) The proliferation of LNCaP and 22RV1 cells is evaluated by EdU assay. (**G**) The colony formation assay shows that PAGln can inhibit the proliferative capacity of LNCaP and 22RV1 cells. (**H**) The Transwell migration assay shows that PAGln can inhibit the migratory ability of LNCaP and 22RV1 cells. Data are presented as the mean ± SD (n = 3). ns P > 0.05, *P < 0.05, **P < 0.01, ***P < 0.001 compared to control, Student's t-test and One-Way ANOVA. PAGln: Phenylacetylglutamine.


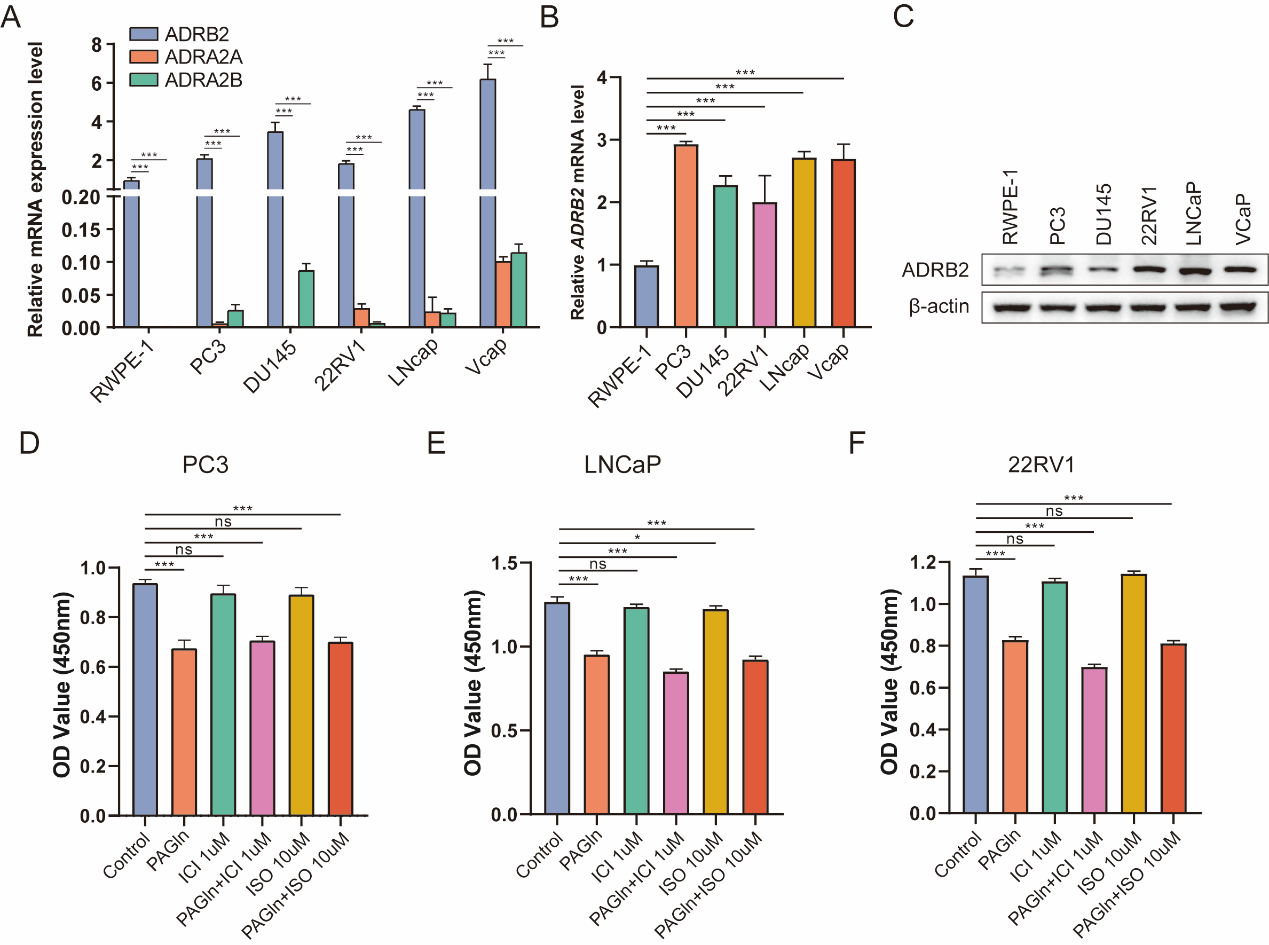


**Supplemental Figure 2.** PAGln's effects on prostate cancer cells are independent of ADRB2. (**A**) The expression levels of mRNA for the adrenergic receptor subtypes ADRA2A, ADRA2B, and ADRB2 in RWPE-1, PC3, DU145, 22RV1, LNCaP, and VCaP cells were detected by q-PCR. (**B, C**) The mRNA and protein expression levels of the ADRB2 in RWPE-1, PC3, DU145, 22RV1, LNCaP, and VCaP using q-PCR and WB assays. (**D- F**) The OD values of PC3, LNCaP, and 22RV1 cells under various treatment conditions were measured using the CCK8 assay, including the control group, PAGln treatment group, ADRB2 inhibitor ICI 118551 treatment group, PAGln combined with ICI 118551 treatment group, ADRB2 agonist ISO treatment group, and PAGln combined with ISO treatment group. Data are presented as the mean ± SD (n = 3). ns P > 0.05, *P < 0.05, **P < 0.01, ***P < 0.001 compared to control, One-Way ANOVA. PAGln: Phenylacetylglutamine. ICI: ICI-118551. ISO: isoproterenol.


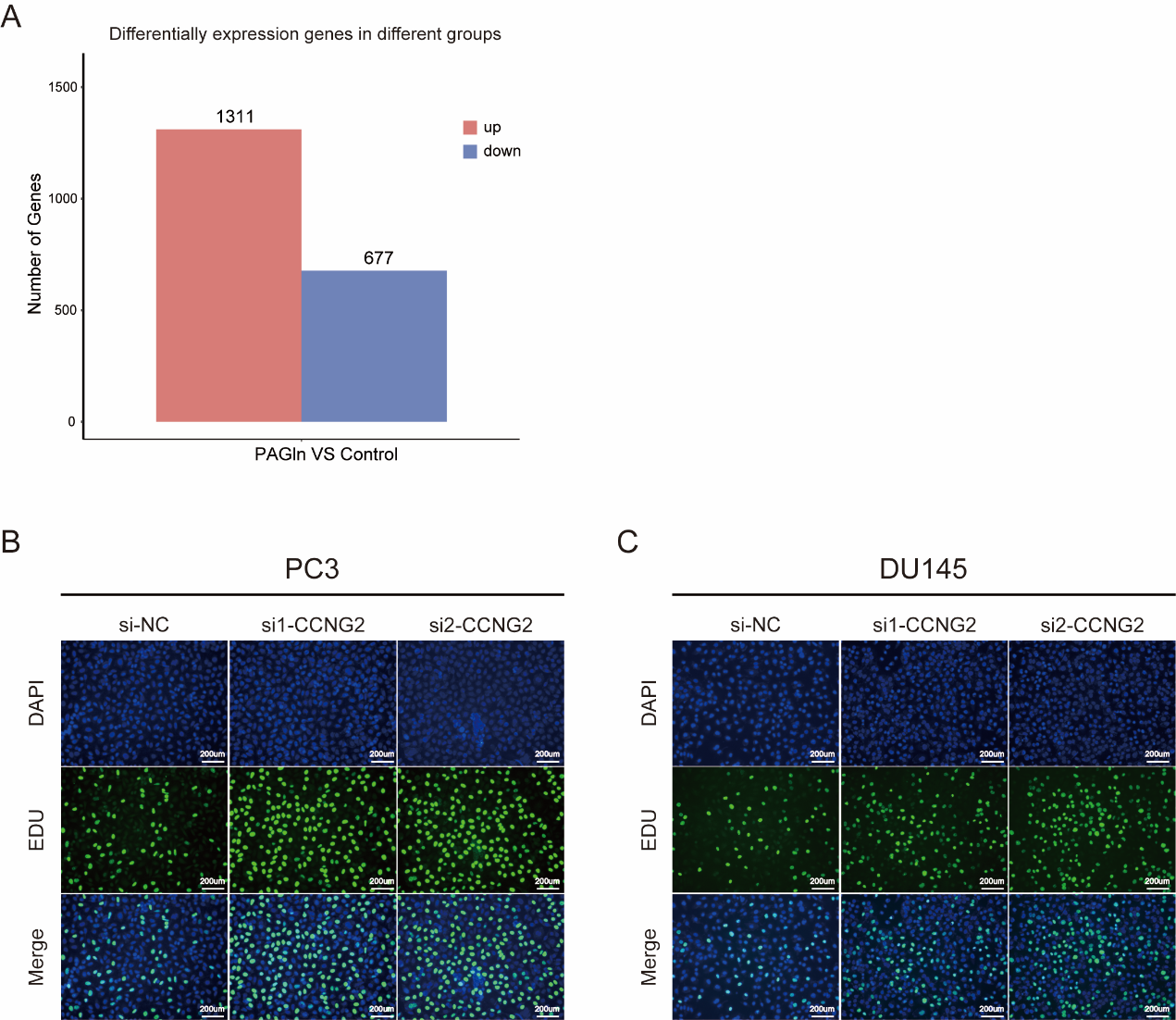


**Supplemental Figure 3**. Knockdown of CCNG2 promotes the proliferation and migration of PCa cells. (**A**) Following a 48-hour intervention with PAGln in PC3 cells, the quantities of upregulated and downregulated differential genes were detected. (**B, C**) The proliferation of PC3 and DU145 cells is evaluated by EdU assay.
