# Supplementary material for: Gut microbiota-derived metabolite phenylacetylglutamine inhibits the progression of prostate cancer by suppressing the Wnt/β-catenin signaling pathway
Source: Front Pharmacol. 2025 Mar 11;16:1528058. doi: 10.3389/fphar.2025.1528058 (PMC11932994; doi:10.3389/fphar.2025.1528058)
Supplement: Supplementary file 3 [file Table2.docx]

**Gut Microbiota-Derived Metabolite Phenylacetylglutamine Inhibits the Progression of Prostate Cancer by Suppressing the Wnt/β-Catenin Signaling Pathway**

**Supplemental legends**

**Supplemental Table 1.** The data from the transcriptome sequencing analysis of PC3 cells of treatment with PAGln and the untreated control group.

**Supplemental Table 2.** The primers used in the present study.

**Supplemental Table 3.** The genes enriched in the P53 signaling pathway among the differentially expressed genes.
