# Supplementary material for: Gut microbiota-derived metabolite phenylacetylglutamine inhibits the progression of prostate cancer by suppressing the Wnt/β-catenin signaling pathway
Source: Front Pharmacol. 2025 Mar 11;16:1528058. doi: 10.3389/fphar.2025.1528058 (PMC11932994; doi:10.3389/fphar.2025.1528058)
Supplement: Supplementary file 5 [file Table6.docx]

**Gut Microbiota-Derived Metabolite Phenylacetylglutamine Inhibits the Progression of Prostate Cancer by Suppressing the Wnt/β-Catenin Signaling Pathway**

**The original data files for Western Blot assay:**

**Fig. 2 G**

| CCNG2 |  |
| --- | --- |
| β-actin |  |

**Fig. 2 I**

| CCNG2 |  |
| --- | --- |
| β-actin |  |

**Fig. 3 B**

| CCNG2 |  |
| --- | --- |
| β-actin |  |

**Fig. 3 D**

| CCNG2 |  |
| --- | --- |
| β-actin |  |

**Fig. 5 A**

|  | PC3 | DU145 |
| --- | --- | --- |
| CCNG2 |  |  |
| p-β-catenin |  |  |
| β-catenin |  |  |
| TCF7 |  |  |
| c-Myc |  |  |
| β-actin |  |  |

**Fig. 5 B**

|  | PC3 | DU145 |
| --- | --- | --- |
| CCNG2 |  |  |
| p-β-catenin |  |  |
| β-catenin |  |  |
| TCF7 |  |  |
| c-Myc |  |  |
| β-actin |  |  |

**Fig. 5 C**

| CCNG2 |  |
| --- | --- |
| p-β-catenin |  |
| β-catenin |  |
| TCF7 |  |
| c-Myc |  |
| β-actin |  |

**Fig. 5 D**

| CCNG2 |  |
| --- | --- |
| p-β-catenin |  |
| β-catenin |  |
| TCF7 |  |
| c-Myc |  |
| β-actin |  |

Fig. 6 I

| CCNG2 |  |
| --- | --- |
| p-β-catenin | 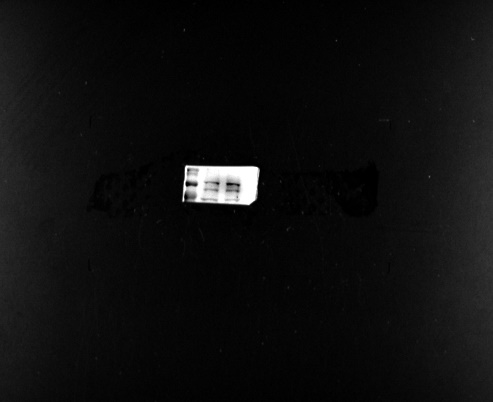 |
| β-catenin |  |
| TCF7 |  |
| c-Myc |  |
| β-actin |  |

**Fig. S2 C**

| ADRB2 |  |
| --- | --- |
| β-actin |  |
